# New-Onset Refractory Status Epilepticus with Claustrum Damage: Definition of the Clinical and Neuroimaging Features

**DOI:** 10.3389/fneur.2017.00111

**Published:** 2017-03-27

**Authors:** Stefano Meletti, Giada Giovannini, Giuseppe d’Orsi, Lisa Toran, Giulia Monti, Rahul Guha, Andreas Kiryttopoulos, Maria Grazia Pascarella, Tommaso Martino, Haris Alexopoulos, Martha Spilioti, Jana Slonkova

**Affiliations:** ^1^Department of Biomedical, Metabolic, and Neural Sciences, Center for Neurosciences and Neurotechnology, University of Modena and Reggio Emilia, Modena, Italy; ^2^Neurology Unit, NOCSAE Hospital, AOU Modena, Modena, Italy; ^3^Clinic of Nervous System Diseases, University of Foggia, Riuniti Hospital, Foggia, Italy; ^4^Department of Neurology, University of Virginia, Charlottesville, VA, USA; ^5^1st Department of Neurology, Aristotle University of Thessaloniki, AHEPA Hospital, Thessaloniki, Greece; ^6^Department of Pathophysiology, Medical School, University of Athens, Neuroimmunology Unit, Athens, Greece; ^7^Clinic of Neurology, University Hospital Ostrava, Ostrava, Czech Republic

**Keywords:** new-onset refractory status epilepticus, claustrum, fever, status epilepticus, refractory status epilepticus, epilepsy

## Abstract

New-onset refractory status epilepticus (NORSE) is a rare but challenging condition occurring in a previously healthy patient, often with no identifiable cause. We describe the electro-clinical features and outcomes in a group of patients with NORSE who all demonstrated a typical magnetic resonance imaging (MRI) sign characterized by bilateral lesions of the claustrum. The group includes 31 patients (12 personal and 19 previously published cases; 17 females; mean age of 25 years). Fever preceded status epilepticus (SE) in 28 patients, by a mean of 6 days. SE was refractory/super-refractory in 74% of the patients, requiring third-line agents and a median of 15 days staying in an intensive care unit. Focal motor and tonic–clonic seizures were observed in 90%, complex partial seizures in 14%, and myoclonic seizures in 14% of the cases. All patients showed T2/FLAIR hyperintense foci in bilateral claustrum, appearing on average 10 days after SE onset. Other limbic (hippocampus, insular) alterations were present in 53% of patients. Within the personal cases, extensive search for known autoantibodies was inconclusive, though 7 of 11 patients had cerebrospinal fluid lymphocytic pleocytosis and 3 cases had oligoclonal bands. Two subjects died during the acute phase, one in the chronic phase (probable sudden unexplained death in epilepsy), and one developed a persistent vegetative state. Among survivors, 80% developed drug-resistant epilepsy. Febrile illness-related SE associated with bilateral claustrum hyperintensity on MRI represents a condition with defined clinical features and a presumed but unidentified autoimmune etiology. A better characterization of *de novo* SE is mandatory for the search of specific etiologies.

## Introduction

Status epilepticus (SE) is the second most common neurologic emergency (1). Up to 40% of SE cases are refractory status epilepticus (RSE) to first- and second-line treatments (2, 3). New-onset RSE (NORSE) is a rare but challenging condition, characterized by the occurrence of a prolonged period of refractory seizures with no identifiable cause in otherwise healthy individuals (4–6). Gaspard et al. (7) reported a large retrospective case-series of 130 NORSE cases evaluated between 2008 and 2013. In some patients, an autoimmune or paraneoplastic etiology was identified but more than half of the cases remained cryptogenic. Poor outcomes were observed in 62% of patients, while 22% of patients died. Therefore, improved knowledge of the electro-clinical features of NORSE, as well as identification of the underlying etiologies and best treatment options are mandatory. Anecdotal evidence suggests that immune-modulating therapies may be effective in SE (8) and in NORSE (9–11).

We recently described a small group of patients who developed NORSE several days after a febrile illness, all with a notable magnetic resonance imaging (MRI) sign characterized by T2 signal alterations involving the bilateral claustrum (12). Several similar cases had previously been described, including several children with similar neuro-radiologic features, which were attributed to the spectrum of febrile infection-related epilepsy syndrome (FIRES) (13–15). Bilateral claustrum abnormalities were associated with medically intractable SE, focal motor seizures, and myoclonic seizures. Diagnostic studies did not reveal an etiology in these cases.

This study aims to organize and better characterize the clinical features of patients with NORSE and T2/FLAIR hyperintense foci in the bilateral claustrum on brain MRI by systematically reviewing the literature and describing newly reported personal cases. Increased awareness of this entity will be relevant in the management of patients with refractory SE.

## Materials and Methods

Information including demographic data, clinical features, diagnostic findings, therapeutic interventions, and clinical outcomes of patients fulfilling the following inclusion criteria were acquired: (a) previously healthy adults (>16 years of age) with refractory SE; (b) MRI evidence of bilateral hyperintense signal alteration of the claustrum on FLAIR/T2 imaging; and (c) no evidence of infectious agents in cerebrospinal fluid (CSF). Exclusion criteria were a previous history of seizures (febrile or afebrile), or a previous or current neurological disorder.

Patients’ data for cases 1–6 were collected and described in a previous publication (12), while for cases 7–12, data were collected during the last 12 months.

Being a retrospective case collection, ethical approval was not required for this study in accordance with the national and institutional guidelines. Scientific advisory boards of participating institutions approved the study according to local regulations. Written informed consent was obtained from patients (for those surviving the SE), by their parents if underage, or by relatives in case of death during the acute phase. The three patients whose video we have included, as supporting information, gave their specific written consent for the brief video sequence of their typical seizures.

### Clinical and EEG Evaluation

Data were collected retrospectively from the participating centers reviewing clinical charts, EEG, and video-EEG recordings (when available). RSE was defined as a SE episode that continues, regardless of the delay since the onset of the seizure, after failure of a trial of at least intravenous benzodiazepines and at least one AED, appropriately chosen and at adequate dosage. Thus, it required admission to the Intensive Care Unit (ICU) and the application of anesthetic drug therapy. Super-RSE (SRSE) was defined as SE that continues or recurs at least 24 h from the beginning of anesthetic therapy or recurs during the reduction or withdrawal of anesthesia (16).

### Laboratory Investigations

All patients were examined for viral and bacterial infections. Viral DNA polyemerase chain reaction tests included HSV 1 and 2, HIV, varicella-zoster virus, HHV 6, cytomegalovirus, Epstein–Barr virus, west nile virus, rubella, parvovirus B19, enterovirus, and mumps. Presence of oligoclonal IgG bands (OB) was assessed by means of isoelectro-focusing, performed with agarose gel support. A panel of autoimmune antibodies, including antinuclear antibodies, anti-phospholipid antibodies, anti-DNA antibodies, anti-cardiolipin antibodies, anti-extractable nuclear antigen antibodies, and anti-thyroid antibodies were analyzed with immuno-enzymatic tests and indirect immuno-fluorescent staining.

Computed tomography of the thorax and abdomen was performed in all patients. An extensive blood analysis for classical onco-neural antibodies (anti-GAD, anti-Yo, Ri, Hu, anti-Ma2) was performed in all subjects, as well as testing for antibodies to *N*-methyl-d-aspartate receptor (NMDAR) (17), leucine-rich glioma inactivated protein 1 (LGI1), and contactin-associated protein-like 2 (Caspr2) (previously attributed to voltage-gated K channels) (18, 19).

### Brain MRI

Patients underwent brain MRI studies with a field intensity of 1.5 or 3 T during the acute phase and follow-up in the chronic phase of the illness. The median number of MRI per patient was four.

### Literature Review

Searches for identification of studies were run from 1990 to 2016 in MEDLINE and PubMed. Searches were limited from 1990 to the present day since studies carried out previously would necessarily have included participants without MRI. The search keywords were: “FIRES”; “NORSE”; “status epilepticus” AND “claustrum”; “epilepsy” AND “claustrum”; “status epilepticus” AND “neuroimaging.” For each citation considered, the abstract was read (when available). The bibliography of each of the retrieved papers was examined to identify relevant references that could have been missed by electronic search. Only peer-reviewed original articles providing images of the claustrum involvement were accepted for inclusion in this article.

## Results

Nineteen cases were retrieved from previous case-reports and series (13–15, 20–32), and 12 personal cases fulfilled the inclusion criteria. Tables 1 and 2 summarize the main clinical and MRI findings of all 31 patients. Detailed descriptions of these cases can be found in the Supplementary Material (Tables S1–S3 in Supplementary Material).

**Table 1:** **Clinical and outcome data of personal and literature cases**.

Reported cases (*n*)	31
**Demographic data**	
Mean age (years ± SD)	24.6 ± 12.4
Age range (years)	7–65
Female gender (%)	55
**Prodromal phase**	
Presence of fever (% of pts)	100
Interval between fever onset and SE onset (days ± SD)	6.1 ± 2.5
Altered mental status (% of pts)	63
Isolated seizures	27
**Acute phase**	
Seizures’ semiology (% of pts)	
Focal motor/s generalization	90
Complex partial	14
Myoclonus/e.p.c.	14
Response to treatments (% of pts)	
Responsive SE	26
Refractory SE	32
Super-refractory SE	42
Patients needing ICU (%)	74
Immuno-modulating therapies (% of pts)	
IV steroids	59
IV Ig	48
PEX	11
None	26
Undetermined	12.9
**Outcomes**	
Death in acute phase (% of pts)	7
Death in chronic phase (% of pts)	4
Chronic epilepsy (% of pts)	78
Cognitive deficits (% of pts)	74
Behavioral deficits (% of pts)	32

*SE, status epilepticus; pts, patients; e.p.c., epilepsia partialis continua; ICU, intensive care unit; IV, intravenous; Ig, immunoglobulin; PEX, plasma exchange*.

*Responsive SE indicates a response to first- and second-line antiepileptic drugs; refractory SE indicates patients who fail to respond to antiepileptic drugs requiring third-line agents (propofol, barbiturate anesthesia). Super-refractory SE indicates cases in which seizures recurred after one cycle of anesthetic treatment*.

**Table 2:** **Magnetic resonance imaging (MRI) findings of personal and literature cases**.

Reported cases (*n*)	31
**MRI**	
Interval between fever onset and claustrum sign (days ± SD)	15.6 ± 7.2
Interval between SE onset and claustrum sign (days ± SD)	10 ± 6.4
Range between SE onset and claustrum sign (days)	3–25
Involvement of other structures (% of pts)	
Medial temporal lobe	33
Thalamus (pulvinar)	3
Cortex (insula)	27

*SE, status epilepticus; pts, patients*.

### Representative Patients

#### Case # 11

**Figure 1:**
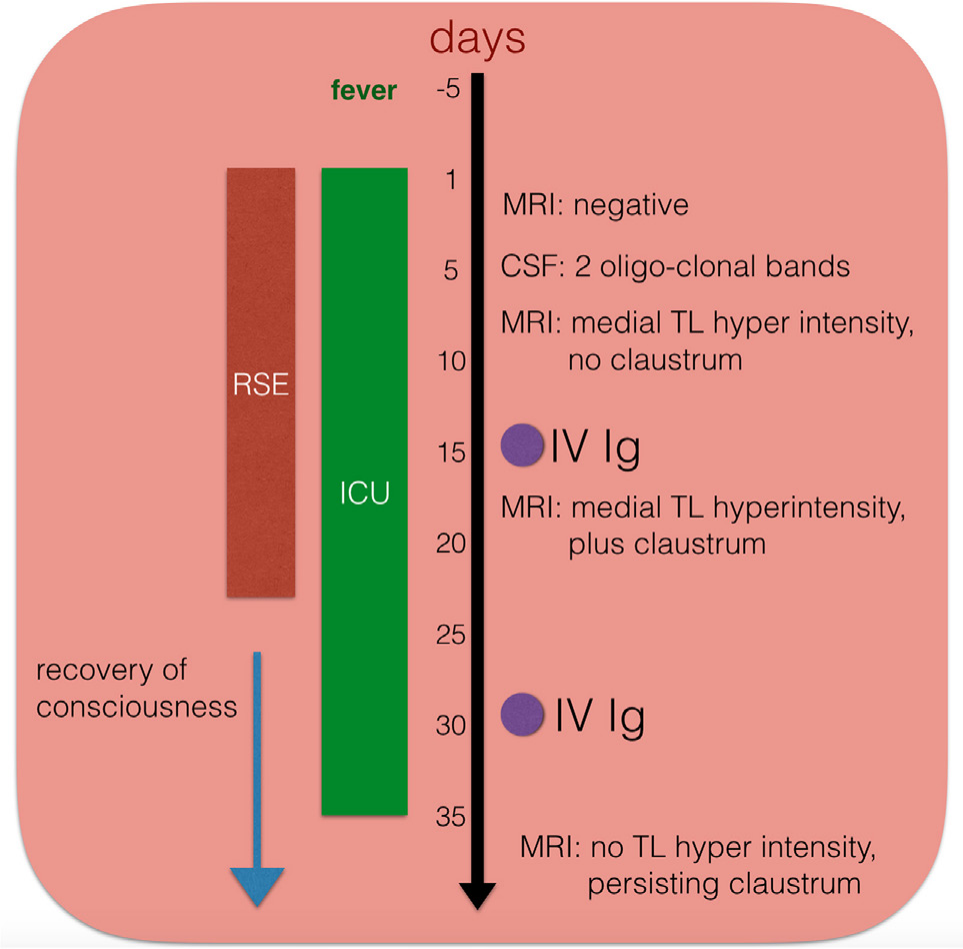
**Schematic temporal evolution, procedures, and immuno-modulating treatment in one patient (case # 11)**. IV Ig, intravenous immunoglobulins; CSF, cerebrospinal fluid; ICU, intensive care unit; RSE, refractory status epilepticus.

A 33-year-old woman presented to the emergency department with worsening confusion over 6 days in the setting of fever and malaise. Within hours of hospital admission she developed focal motor status with right facio-brachial clonic seizures with secondary generalization, originating from the left hemisphere electrographically. First- and second-line antiepileptic agents failed to control seizures, and she was transferred to the ICU. She developed independent seizures originating from the left and right hemispheres, clinically characterized by facial clonic twitching. During the acute phase of the disorder, she was treated with lacosamide, phenytoin, levetiracetam, and phenobarbital. Propofol and midazolam were used as third-line agents. She also received two cycles (5 days each) of intravenous immunoglobulins (IVIG). Third-line agents were successfully weaned-off and, after 20 days in the ICU, the patient was finally discharged to the neurology unit and then home. Despite medication adjustments, with ultimate treatment with lacosamide 400bid and phenytoin 300bid, she developed a chronic drug-resistant focal epilepsy with weekly seizures, and readmissions for breakthrough seizures. Four months after presentation with SE, the patient showed deficits in short-term verbal and visuospatial memory, learning difficulties, and executive function problems. At 6 months she returned to her normal life with family support. Figure 1 is a schematic representation of the patient journey through the SE (see also Video S1 in Supplementary Material).

#### Case # 8

A 25-year-old man was admitted for evaluation of his first generalized tonic–clonic seizure after 5 days of fever, malaise, and headache. He soon developed very frequent, refractory focal seizures, originating either from the right (more commonly) or the left (less commonly) hemisphere. When the seizures where clinically observed, as many of them were subclinical, he demonstrated behaviors of confusion, staring, oral and hand automatisms, and occasional head version. Seizures were refractory to first- and second-line AEDs (including fosphenytoin, lacosamide, levetericetam, valproic acid, phenobarbital, and clonazepam), so he received third-line agents (propofol, midazolam, and ketamine) in the ICU. He was treated with plasma exchange (five treatments over 9 days), finally resulting in an electro-clinical improvement. After a total of 30 days in the hospital, with 17 of those in the ICU, he was discharged to a rehabilitation facility. On discharge his neurological examination showed mild gait ataxia, limb dysmetria, with intermittently perseverative speech and hyper-religiosity. He was readmitted several times over the next year for breakthrough generalized tonic–clonic seizures or clusters of dyscognitive seizures described as behavorial arrest, leftward head deviation and dystonic posturing of his left arm. EEG did not identify a consistent focus of onset for his seizures (at times bifrontal, right temporal, or left fronto-temporal). A fluorodeoxyglucose PET brain scan could not localize an epileptogenic focus. His MRI performed at 12 months from presentation demonstrated unchanged T2/FLAIR hyperintense foci in the cerebral white matter in bilateral claustrum and external capsules, as well as progressive diffuse cerebral volume loss. Despite many medication adjustments, the patient developed a chronic drug-resistant epilepsy, with his most recent antiepileptic regimen (listed in total daily doses) including clonazepam 6 mg, lacosamide 400 mg, levetiracetam 3,000 mg, phenobarbital 200 mg, valproic acid 3,500 mg. The overall seizure frequency did not improve on the modified Atkins diet. Subsequent treatment for refractory seizures with IVIG on two occasions, 1 month and again 3 months after his discharge for SE, did not result in clinical improvement. Ultimately at 1 year after initial presentation, he reported at least one focal dyscognitive seizure a week but had restarted physical activities such as weight lifting and swimming. Memory issues and daytime somnolence due to medications limited his ability to return to employment.

### The Prodromal Phase

The patients affected by this condition are generally young (mean age at SE onset is 25 years; mode 29 years). A prodromal phase of fever with mild flu/cold symptoms or general malaise preceded SE by 6 days on average (ranging from 2 to 14 days) in all patients for whom we have these data (information is missing in 3 cases).

### The Acute Phase

In 63% of the reported cases, patients presented to the emergency room for sudden onset altered mental status, ranging from mild fluctuations in mental status to stupor. This could appear either as the only symptom (21 cases) or in combination with seizures (1 case), while 27% of the patients (8 patients) presented with seizures only. In the early days, all personal cases were treated with antiviral and antibacterial drugs in the possibility of an infectious cause of the clinical picture and waiting for the laboratory results (negative in all patients, see Materials and Methods) on blood and CSF for infectious agents.

In regards to seizure semiology, 90% of patients had focal motor or tonic–clonic seizures, while 14% of patients had isolated complex partial seizures. One of the most striking characteristics in these cases is the extremely rapid evolution of seizure during the acute phase. For example the majority of patients presented with bilateral motor seizures (focal hemiclonic or multifocal myoclonic seizures with alternating affected side), which then could quickly evolve into a generalized non-convulsive or subtle/myoclonic SE. See supplementary videos (Video S1 in Supplementary Material, from case # 11; Video S2 in Supplementary Material, from case # 1; Video S3 in Supplementary Material, from case # 7). Overall, 74% of patients rapidly developed a RSE/SRSE (RSE: 32%; SRSE 42%), requiring admission to the ICU and anesthesia administration soon after initial presentation.

#### MRI Findings

**Figure 2:**
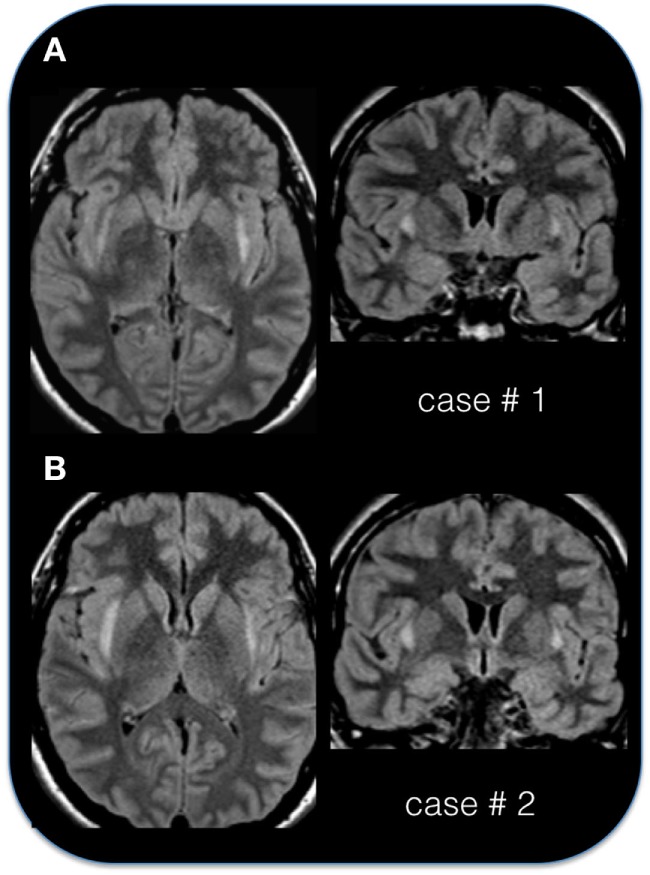
**The “claustrum sign” in two patients**. **(A)** Axial and coronal FLAIR sequence in case # 1, 29-year-old female, acquired 10 days after status epilepticus (SE) onset. **(B)** Axial and coronal FLAIR sequence in case # 2, 24-year-old female, acquired 4 days after SE onset. Note the hyperintense appearance of the capsula esterna/claustrum region in the two cases.

**Figure 3:**
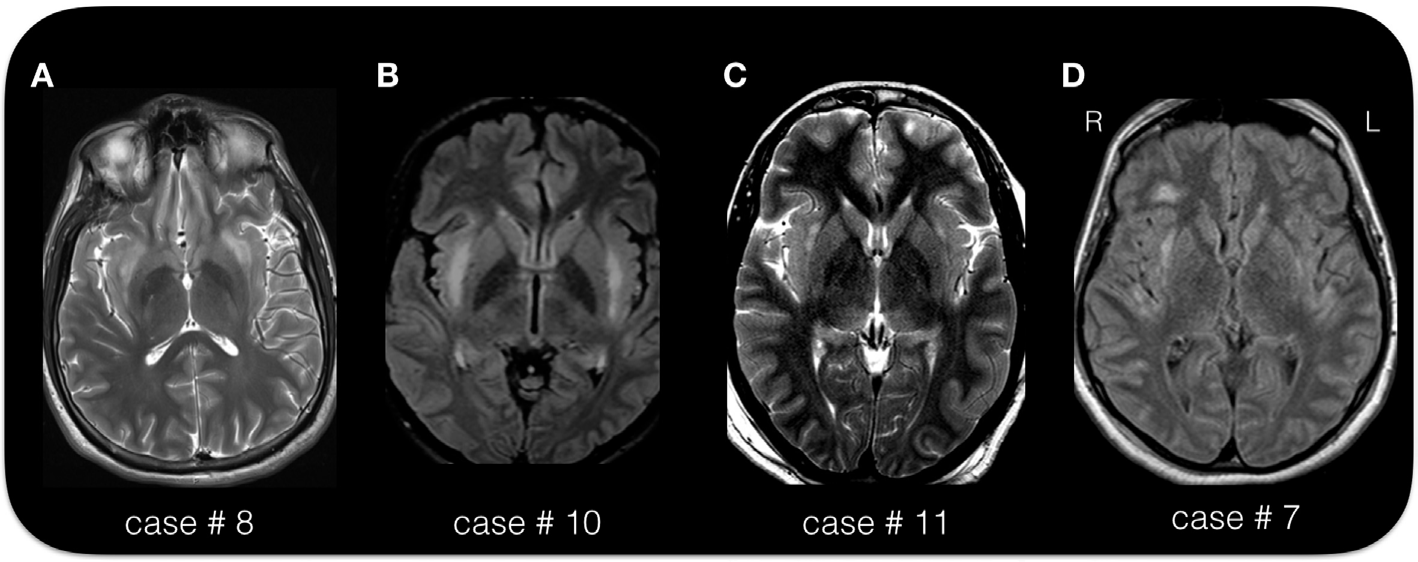
**The “claustrum sign” in four patients**. **(A)** Axial T2 sequence in case # 8, 25-year-old male, acquired 15 days after status epilepticus (SE) onset. **(B)** Axial FLAIR sequence in case # 10, 19-year-old female, acquired 6 days after SE onset. **(C)** Axial T2 image in case # 11, 33-year-old female, acquired 21 days after SE onset. **(D)** Axial FLAIR image in case # 7, 38-year-old female, acquired 25 days after SE onset. Note the hyperintense appearance of the capsula esterna/claustrum region in the four cases. Despite the different MRI scanner and image parameters, the hyperintensity of the claustrum is easily recognizable in each case. The exact location and extension of the signal alteration varies across the four cases. In all patients is present a bilateral involvement. Note the signal alteration extending to the insular/peri-insular cortex and subcortical white matter in panel **(D)**.

As per inclusion criterion, the MRI during the acute phase, showed bilateral symmetric or asymmetric changes of the claustrum (so called “claustrum sign”) that appeared hyperintense on T2-weighed images (see Figures 2 and 3). On average, the claustrum sign was observed 10 days after SE onset (ranging from 3 to 25 days). This finding was isolated to the claustrum in 13 patients (43% of the cases), or it was part of a more extended signal hyperintensity alteration together with other limbic areas (see Table 2), including the insula (5 cases), the medial–temporal lobe (8 cases), the insula and medial–temporal lobe at the same time (2 cases), or the insula and other more diffuse cortical areas (1 case). The alteration involving the medial temporal region consisted in bilateral hyperintensity of the amygdala/hippocampus. In one case, the bilateral claustrum was seen together with a unilateral pulvinar hyperintensity.

For the 12 newly described personal cases, a follow-up MRI study was obtained at various points after resolution of SE. In all but one case, the claustrum alterations disappeared after resolution of SE, and in one case the claustrum sign disappeared during ongoing SE. Follow-up MRI showed a variable degree of atrophy, ranging from very mild to severe and diffuse. Bilateral volume reduction of the hippocampus was present in four cases, always concomitantly with cortical atrophy.

#### EEG Findings

**Figure 4:**
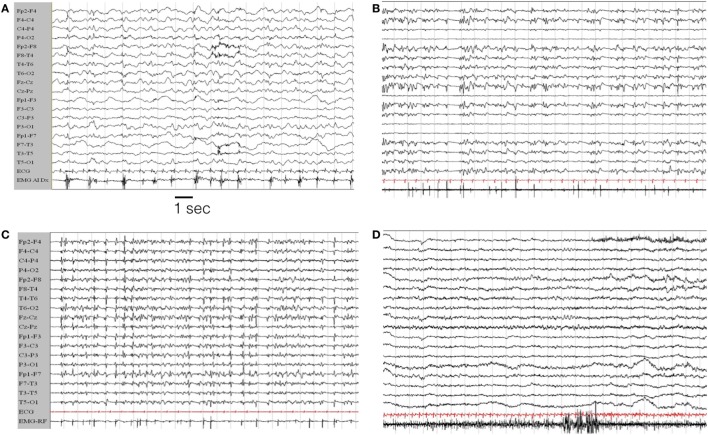
**EEG evolution in case # 1**. **(A)** EEG during the initial hours of status epilepticus (SE). The patient was awake but confused with sub-continuous myoclonic jerks involving predominantly left limbs. The last channel records the surface EMG activity from the left anterior tibialis muscle. The EEG traces show a general slowing of the background activity, more evident on the right hemisphere, where periodic sharp complex are evident, often time-locked with myoclonic jerks. **(B,C)** EEG during the evolution in refractory SE, in the from of a myoclonic SE. The patient is under general anesthesia with barbiturates plus antiepileptic drugs poly-therapy. **(D)** Represents the EEG evolution during recovery. EEG recording parameters: high-pass filter: 0.3 Hz; low-pass filter 50 Hz. EMG parameters: high-pass filters 50 Hz; low-pass filter 250 Hz. Sampling frequency 1,024 Hz.

**Figure 5:**
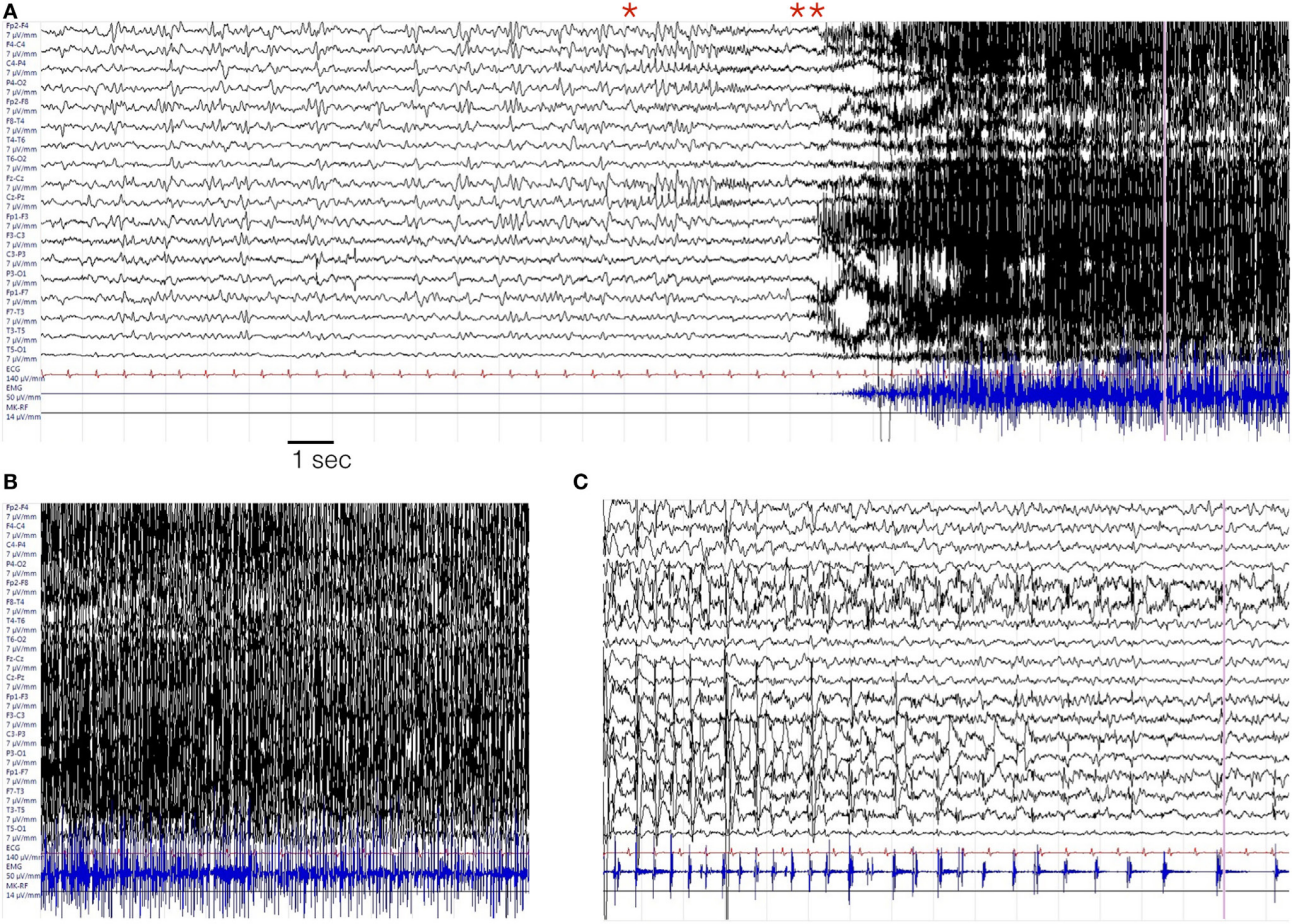
**Representative seizure in case # 7**. **(A)** Periodic theta burst involving the right fronto-temporal leads evolving into a right ictal rhythmic discharge (*) and then in a diffuse EEG “flattening” (**). **(B)** Diffuse EMG artifacts during the tonic–clonic phase of the seizures. **(C)** Terminal part of the seizures with predominant involvement of the left fronto-temporal region. In each panel, the EMG trace is recorded from the orbicularis oris muscle. EEG recording parameters: high-pass filter: 0.3 Hz; low-pass filter 50 Hz. EMG parameters: high-pass filters 50 Hz; low-pass filter 250 Hz. Sampling frequency 1,024 Hz.

**Figure 6:**
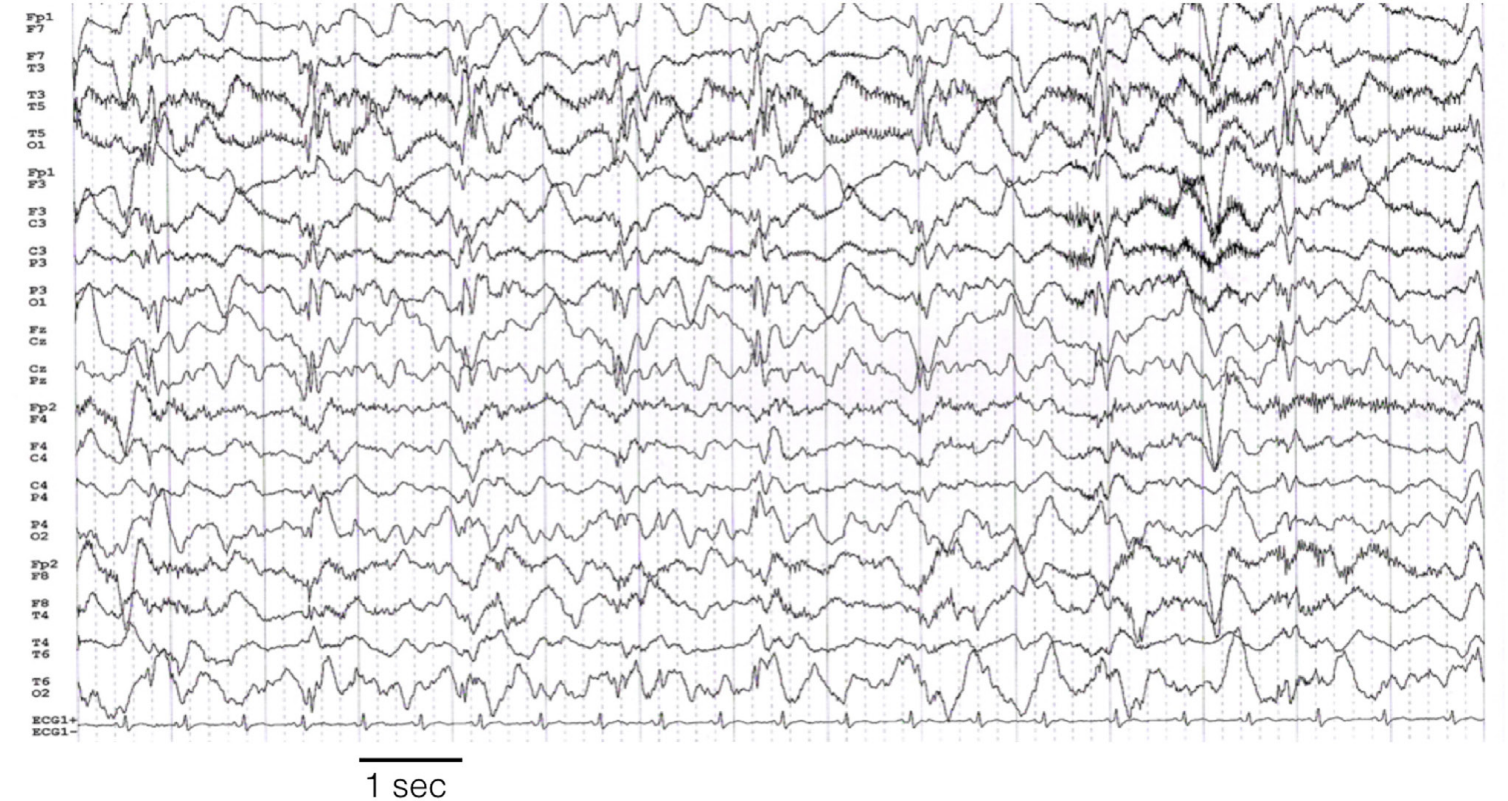
**Left hemisphere periodic discharges in case # 10**. EEG recording parameters: high-pass filter: 0.3 Hz; low-pass filter 30 Hz. Sampling frequency 128 Hz.

The characteristics of the EEG are defined only for the 12 personal cases. Eight out of 12 patients were monitored by continuous EEG (cEEG; meaning a continuous EEG recording of longer duration of 8 h per day). In four subjects, shorter and periodic EEG recordings were performed on clinical needs. On average, patients were monitored for 22 days (range: 3–90 days). Seizures were recorded in each case (>100 in seven patients; >20 in five). The most striking pattern was the presence of periodic discharges (PD) (either generalized, bilateral independent, or lateralized). Often in different period of SE evolution the pattern changed so that different types of PD were observed in each case. Generalized PD were observed in five cases; lateralized unilateral PD were present in five; bilateral independent or multifocal PD were observed in 11 cases (see Figures 4–6).

#### Laboratory Investigations

Cerebrospinal fluid samples are available in 11 of the personal cases. A mild lymphocytic pleiocytosis (5–40 cells) was present in 7 cases and oligo-clonal bands were observed in three. Among all these cases, the CSF autoantibodies panel was negative (see Table S2 in Supplementary Material) and the etiology remains cryptogenic. Among the previously published cases, one had positive anti-VGKC antibodies and in two cases there were signs of a recent virus infection (mumps in one case and HSV1 in the other one) without any sign of a direct CNS involvement.

#### Immune-Modulating Treatments

Besides antiepileptic drugs and anesthetic therapy, 20 out of 27 patients (74%) received immunotherapy (for five literature cases, these data are lacking). High dose intravenous steroids were used in 59% of the patients; intravenous immune-globulins were used in 48% of patients, and plasma exchange in 11%.

### The Chronic Phase and Outcomes

Two patients died during their initial hospitalization. Another patient died 2 years later, and his death was defined as a probable sudden unexplained death in epilepsy. Eighteen out of 23 patients (78%) developed a chronic focal epilepsy, mostly resistant to medical therapy. Moreover, 65% of the patients developed cognitive deficits. These deficits were reported to be mild for 60% and moderate to severe for 40% of these patients. Finally, 32% of patients ultimately were left with behavioral alterations (impulsivity, psychosis, episodes of aggression, anxiety). Overall, only 32% of the patients returned to their clinical baseline condition.

## Discussion

New-onset refractory status epilepticus represents a challenging entity. Only in recent years, it has begun to shed light on these cases, showing that a significant portion has an autoimmune and/or paraneoplastic etiology. The aim of this report is an attempt to define a group of patients that we believe share a constellation of symptoms, EEG, and MRI findings. In 2015, we previously described six patients presenting with a refractory SE after a febrile illness and a striking signal alteration involving the region of the claustrum on brain MRI (12). In last 12 months, we collected new cases with similar clinical histories and imaging findings (“the claustrum sign”). The claustrum sign was associated with an aggressive refractory form of SE. The temporal evolution of the clinical picture was very well defined. First, patients experienced a prodromal phase characterized by fever and altered mental status, followed by an acute phase with focal motor, tonic–clonic, and myoclonic seizures, often with refractory SE requiring ICU admission and anesthetic drugs, followed by a chronic phase with the development of a focal drug-resistant epilepsy in 80% of surviving patients. The T2/FLAIR hyperintensity of the claustrum/capsula externa region was bilateral, though sometimes asymmetric, in every case, and in half of the cases extended into the insular cortex and the medial temporal lobe region as well. We confirmed that these alterations follow the onset of the status by an average of 10 days and resolved over the followings weeks in the majority of patients. It is unclear if these findings are a consequence of ictal activity or a reflection of a pathogenic mechanism affecting the same bilateral brain networks. All but one of the patients experienced complete resolution of the MRI abnormalities, with one patient demonstrating resolution during ongoing SE, while one patient showed the persistence of hyperintense foci on a 12-month follow-up MRI. Probably, only future prospective studies, with the use of MRI at standardized time-points respect with SE onset, will be able to elucidate the role and mechanisms underlying this imaging sign.

Regarding, the possible etiology of this condition we were unable to identify a specific causative antibody for known autoimmune syndromes. CSF samples showed lymphocytic pleiocytosis and oligo-clonal bands, which supports a role for the immune system in the pathophysiology of this condition. Additionally, patient improvement with immunomodulatory treatment could support this hypothesis. Beside this speculative consideration, it is, however, important to recognize that at present no definite etiology was reliably demonstrated, and even if we excluded several possible infectious conditions, the possibility of an unknown infectious agent, as causative of the reported NORSE cases cannot be completely ruled-out. More remote seems the possibility of drug intoxication for the presented patients: no one had a history of drug abuse or psychiatric comorbidities; no one had possible exposure to possible environmental toxic substances. Given that, we agree with previous literature that as for the actual knowledge (and its limits) it is advisable to rapidly treat these patients with immune modulatory agents, beyond just the standard treatment of SE (7, 9–11).

From the electro-clinical point of view, a very aggressive form of SE was observed in the majority of the patients. The physiological properties of the claustrum can be important to explain this particular evolution. Indeed, the claustrum could be a key region in promoting the propagation and synchronization of abnormal epileptic activity from several cortical regions (12, 32). Two properties of claustrum neurons are relevant: (a) claustro-cortical fibers connect the claustrum with several cortical areas including the prefrontal, pre-postcentral, orbitofrontal, and medial temporal cortex (33, 34); (b) the claustrum is constituted by densely packed and tightly interconnected GABAergic interneurons whose damage could promote a state of hyper-synchronization and bindings of activity from several distant cortical regions (34–38). Notably, these experimental findings can account for the frequently observed presence of PD over one or both hemispheres during continuous EEG monitoring in our patients, as well as the often bi-hemispheric or independent seizure origin during the acute phase. Moreover, the involvement of the claustrum/insular cortex explains also the frequent involvement of the oro-facial muscles (see videos in Supplementary Material). Indeed, the involvement of this body region is not surprising considering a probable ictal involvement of the adjacent fronto-opercular cortex, controlling the oro-facial movements.

Finally, concerning the outcomes and the chronic phase, we confirmed that a very high proportion (about 80%) of the patients developed a chronic epilepsy. In this respect, we cannot say if epilepsy during the chronic phase is the consequence of an epileptogenic process related to the SE or rather if chronic epilepsy is the consequence of a persistently “active” etiological factor. Surely, in the majority of the patients, the epilepsy observed during the chronic phase was drug-resistant, with multiple seizures per month. Notably, one of the patients died 2 years after SE termination due to cardio-respiratory arrest after a suspected tonic–clonic seizure during sleep.

### Conclusion

Febrile illness-related SE with claustrum hyperintensity represents a condition with defined clinical features. We hope that future studies will help to better understand the pathophysiology and the etiology of this condition that at present remains speculative, though a probable autoimmune mechanism is suspected. Prospective, multi-centric, studies on NORSE are mandatory to answer several open questions on this condition.

## Author Contributions

SM, GG, and JS: study design, data collection, and drafting and revising the manuscript. LT, RG, GO, and GM: data collection and revising the manuscript. AK, MP, TM, HA, and MS: data collection and interpretation. All the authors contributed to discussion of the results and read and approved the final version of the manuscript.

## Conflict of Interest Statement

None of the authors has any conflict of interest to disclose. We confirm that we have read the Journal’s position on issues involved in ethical publication and affirm that this report is consistent with those guidelines. SM received Research grant support from the Ministry of Health (MOH) and from the non-profit organization CarisMo Foundation and also has received personal compensation as scientific advisory board member for UCB and EISAI. Other authors report no disclosures.

## Supplementary Material

Video S1 **Video sequence from case # 11 showing clonic seizures involving the face while staying in intensive care unit and before starting third-line treatment with propofol**.

Video S2 **Video sequence from case # 1 showing the impending status with repetitive myoclonia involving the trunk and lower limb while awake**. The patient is partially oriented and reports intense fear and discomfort. The second sequence shows high-frequency erratic lower limbs myoclonus during the evolution in refractory status.

Video S3 **Video sequence of sub-continuous facial clonic movements from case # 7 while in the intensive care unit and during a weaning failure from anesthetics**.

## Abbreviations

AED, antiepileptic drugs; AERRPS, acute encephalopathy with repetitive refractory partial seizures; CSF, cerebrospinal fluid; FIRES, febrile infection-related epilepsy syndrome; FLAIR, fluid attenuation inversion recovery; ICU, intensive care unit; LGI1, leucine-rich glioma inactivated protein 1; NMDAR, *N*-methyl-d-aspartate receptor; NORSE, new-onset refractory status epilepticus; PD, periodic discharges; SE, status epilepticus; RSE, refractory status epilepticus.
