# Supplementary material for: New-Onset Refractory Status Epilepticus with Claustrum Damage: Definition of the Clinical and Neuroimaging Features
Source: Front Neurol. 2017 Mar 27;8:111. doi: 10.3389/fneur.2017.00111 (PMC5366956; doi:10.3389/fneur.2017.00111)
Supplement: Supplementary file 1 [file Table_1.DOCX]

**Supplementary Table 1. Clinical data of personal cases.**

| **Pt.** | **Age/Gender/Ethnicity** | **Fever (days)** | **Presenting symptoms** | **Seizures semiology** | **Frequency of seizures** | **Evolution of SE** | **Need of ICU** | **AED used** | **Other Drugs** | **Barbiturate Coma (Days)*** | **other therapy** | **Clinical outcome** | **Seizure outcome** |
| --- | --- | --- | --- | --- | --- | --- | --- | --- | --- | --- | --- | --- | --- |
| 1 | 29/F/Caucasian | 5 | Confusion/  stupor | Staring/eye deviation and focal hemiclonic; then generalized myoclonic state | Uncountable | SRSE | Yes | PHT, LEV, LCM | MDZ, Propofol | Yes (10) | SteroidsIVIG | Normal life; mild deficits in executive functions | Focal epilepsy on LEV, CLB |
| 2 | 24/F/Caucasian | 7 | Confusion/  stupor | Focal hemiclonic, myoclonic; then generalized | Uncountable | RSE | Yes | PHT, LEV, TPM, CLB | MDZ, Propofol | No | Steroids | Cognitive and behav. deficits | Focal epilepsy (2-3 s/m) on LEV, CBZ, CLB |
| 3 | 29/F/Asain | 5 | Confusion/  stupor | Focal hemiclonic, myoclonic; then generalized | Uncountable | SRSE | Yes | VPA, PHT, TPM, LEV, LCM, CBZ | MDZ, Propofol | Yes (14) | Steroids | Death | - |
| 4 | 35/M/Caucasian | 6 | Seizures | Eye deviation and focal hemiclonic, with alternating side; then generalized | Uncountable | RSE | Yes | VPA, LEV, PHT | MDZ | No | SteroidsPEX | Normal life, slight attentional deficits but died at 2 years follow-up (SUDEP) | Focal epilepsy (1 s/m) on LTG, LEV |
| 5 | 21/M/Arabian | 3 | Headache/  sleepiness | Focal hemiclonic, with alternating side; then generalized | Uncountable | SRSE | Yes | VPA, PHT, LEV, TPM | MDZ, Propofol | Yes (5) | Steroids IVIG | Normal life | NA |
| 6 | 17/F/Caucasian | 6 | Stupor/severe sleep disruption | Focal motor, tonic, chewing and complex automatisms | Isolated seizures | SRSE | Yes | VPA, CBZ, PB | MDZ, Propofol | Yes (7) | SteroidsIVIG | Mild cognitive deficit, attention deficit | Seizure-free final follow-up |
| 7 | 38/F/Caucasian | 5 | Confusion/  stupor | Generalized, then focal motor - face and UL | Uncountable | SRSE | Yes | LCM, PHT, LEV, PB, PER, VPA, TPM | MDZ, Propofol, Ketamine, | Yes (84) | SteroidsIVIG, PEX | Vegetative state | Focal Epilepsy LCM, LEV, TPM, |
| 8 | 25/M/Caucasian | 5 | Headache/  malaise | Focal, with alternating side; staring, automatism, head version | Uncountable | SRSE | Yes | LCM, PHT, LEV, PB, VPA | MDZ, Propofol, Ketamine | No | PEX | Behavioral impairment (impulsivity, poor judgment, hyper-religiosity) | Focal epilepsy (1-2 s/d) on LCM, LEV, PB |
| 9 | 38/M/Caucasian | 10 | Headache/  malaise/  confusion | Generalized | Isolated seizures | RSE | Yes | PHT, VPA | MDZ | No | IVIG | Behavioral impairment and psychosis with episodes of aggression | Focal epilepsy (1-2 s/m) on LCM, CBZ, VPA |
| 10 | 19 /F/ Caucasian | 4 | Seizures | Generalized, then focal motor - face and UL | Uncountable | RSE | Yes | PHT, LEV, VPA, LCM | MDZ, Propofol | No | Steroids IVIG | Mild memory deficit | Focal Epilepsy LCM, LEV, TPM |
| 11 | 33/F/Caucasian | 5 | Seizures | Focal hemiclonic, then generalized | Uncountable | SRSE | Yes | PHT, LCM, LEV, PB | MDZ,  Propofol, Remifentanil | No | IVIG | Verbal and visuo-spatial memory deficits, attention deficits | Focal epilepsy (3-4 s/w) on PHT, LCM |
| 12 | 24/M/Asian | 6 | Malaise/  confusion | Focal, with alternating side; hemiclonic, then generalized | Uncountable | RSE | Yes | NA | MDZ, Propofol | No | SteroidsIVIG | Normal life | Focal epilepsy seizure free on monotherapy |

PHT: phenytoin; LEV: levetiracetam: LCM: lacosamide; TPM: topiramate; CLB: clobazam; VPA: valproate; PB: phenobarbital; PER: perampanel; MDZ: midazolam; IVIG: intravenous immunoglobulins; PEX: plasmapheresis; RSE: refractory Status Epilepticus; SRSE: Super-Refractory Status Epilepticus; SUDEP: Sudden Unexpected Death in Epilepsy; NA: Not available.
