# Supplementary material for: New-Onset Refractory Status Epilepticus with Claustrum Damage: Definition of the Clinical and Neuroimaging Features
Source: Front Neurol. 2017 Mar 27;8:111. doi: 10.3389/fneur.2017.00111 (PMC5366956; doi:10.3389/fneur.2017.00111)
Supplement: Supplementary file 2 [file Table_2.DOCX]

**Supplementary Table 2. Investigational data in personal cases.**

| **Pt.** | **CSF** | | **EEG** | **Brain MRI in acute phase** | | | | | **Vigilance** | **Other negative findings** |
| --- | --- | --- | --- | --- | --- | --- | --- | --- | --- | --- |
|  | **Cells (ul)** | **Oligo. bands** |  | **Claustrum** | **Other brain regions** | **Days from fever onset** | **Days SE onset** | **Most significant alterations** |  |  |
| 1 | 3 | Absent | G. SW; PD; centro-temporal SW | Bilateral | Insular cortex | 17 | 10 | High-signal in FLAIR and T2 | Coma (on anaesthetic treatment) | LGI1, Caspr2, NMDAr, AMPAr, GABA(B), GABA(A), mGlu-R1-R3-R5, POLG1 mutations |
| 2 | 0 | Absent | G. SW; PD; centro- parietal SW | Bilateral | None | 11 | 4 | High-signal in FLAIR, T2 and DWI with normal ADC | Coma (on anaesthetic treatment) | LGI1, Caspr2, NMDAr, AMPAr, GABA(B), mGlu-R3, |
| 3 | 20 | Absent | PD, SW with alternating side predominance (left> right) | Bilateral | None | 9 | 3 | High-signal in FLAIR, T2 and DWI with normal ADC | Coma (on anaesthetic treatment) | LGI1, Caspr2, NMDAr, |
| 4 | 19 | Absent | PD, SW with alternating side predominance (left> right) | Bilateral | None | 13 | 9 | High-signal in FLAIR, T2 and DWI with normal ADC | Stupor | LGI1, Caspr2, NMDAr, VGCC, mGlu-R3, |
| 5 | 23 | Absent | Multifocal with fronto-temporal, occipital SW | Bilateral | Right posterior thalamus | 10 | 3 | High-signal in FLAIR, T2 and DWI with normal ADC | Coma (on anaesthetic treatment) | LGI1, Caspr2, NMDAr, AMPAr, GABA(B), mGlu-R3 |
| 6 | 10 | Present | Fronto-temporal bilat theta, L or R fronto-temporal SW | Bilateral | None | 10 | 4 | High-signal in FLAIR, T2 and DWI with normal ADC | Coma (on anaesthetic treatment) | LGI1, Caspr2, VGCC-Ab, NMDAr |
| 7 | 4 | Present | G. SW; GPD; fronto-temporal theta | Bilateral (L > R) | insular, temporo, parietal, occipital | 30 | 25 | High-signal in FLAIR, T2 and DWI with normal ADC | Coma (on anaesthetic treatment) | LGI1, Caspr2, NMDAr, AMPAr, GABA(B), GABA(A), mGlu-R1-R3-R5 |
| 8 | 12 - 30 | Absent | Multifocal, bilateral independent seizures; PD | Bilateral | None | 22 | 15 | High-signal in FLAIR, T2 and DWI with normal ADC | Coma (on anaesthetic treatment) | LGI1, Caspr2, NMDAr, AMPAr, GABA(B), mGlu-R3 |
| 9 | 5 | Absent | Fronto-temporal bilat theta, L or R fronto-temporal SW | Bilateral | bilateral hippocampal hyperintensity | 12 | 5 | High-signal in FLAIR, T2 | Coma (on anaesthetic treatment) | LGI1, Caspr2, VGCC-Ab, NMDAr |
| 10 | 4 | Absent | continuous Left LPDs, right SW | Bilateral | None | 12 | 6 | High-signal in FLAIR, T2 | Conscious | LGI1, Caspr2, NMDAr, AMPAr,  mGlu-R1-R3-R5 |
| 11 | 15 - 40 | Present | Right frontal spikes, seizures and PD | Bilateral (R > L) | bilateral hippocampal hyperintensity | 26 | 21 | High-signal in FLAIR, T2 | Vegetative state | LGI1, Caspr2, NMDAr, VGCC, AMPAr, |
| 12 | 5 | Absent | NA | Bilateral | NA | 14 | 10 | High-signal in FLAIR, T2 | Coma (on anaesthetic treatment) | LGI1, Caspr2, NMDAr, VGCC, AMPAr, |

CSF: cerebrospinal fluid; G: generalized; SW: slow waves; PD: periodic discharges; GPD: generalized periodic discharges; LPD: lateralized periodic discharges; L: left: R: right; NA: Not available;
