# Supplementary material for: New-Onset Refractory Status Epilepticus with Claustrum Damage: Definition of the Clinical and Neuroimaging Features
Source: Front Neurol. 2017 Mar 27;8:111. doi: 10.3389/fneur.2017.00111 (PMC5366956; doi:10.3389/fneur.2017.00111)
Supplement: Supplementary file 3 [file Table_3.DOCX]

**Supplementary Table 3. Main clinical findings in previous published cases.**

| **Ref./**  **Year** | **Name given to the condition** | **N. of Pts** | **Age** | **Gender** | **Presenting symptoms** | **Fever (days before onset)** | **Presumed aetiology** | **Seizure semiology** | **Evolution of SE** | **MRI from SE onset (days)** | **Other MRI findings** | **Immuno-therapy** | **Outcome/**  **Epilepsy** | **Consciousness at time of MRI** |
| --- | --- | --- | --- | --- | --- | --- | --- | --- | --- | --- | --- | --- | --- | --- |
| Kimura et al./1994 | None | 1 | 7 | M | Lethargy | 7 | HSV1 (herpes stomatitis the week before) | Focal motor | RSE | 11 | None | None | Behavioural impairment  Chronic epilepsy | Severe encephalopathy, no anaesthesia |
| Sperner et al./1996 | None | 1 | 12 | F | Dizziness, fatigue | 14 | None | Focal motor | Responsive | 7 | None | Steroids | Normal life  No epilepsy | Conscious |
| Nixon et al./ 2001 | None | 1 | 35 | M | Seizures, stupor | 4 | None | Focal motor | SRSE | 13 | Bilateral mesial temporal hyperintensity | None | Death | Comatose state with anaesthesia |
| Shiihara et al./2006 | Acute enc. with refractory SE | 1 | 12 | F | Fluctuation of vigilance | 7 | None | Focal motor | SRSE | 20 | Bilateral mesial temporal hyperintensity then atrophy | None | Severe cognitive- deficit  Chronic Epilepsy | Comatose state with anaesthesia |
| Ishida et al./2006 | Non-herpetic limbic enc. | 1 | 8 | M | Seizures | Und. | None | Und. | Responsive | Und. | right hIppocampus | Und. | Und. | Und. |
| Saito et al./2007 | AERRPS | 1 | 10 | M | Fluctuation of vigilance | 6 | None | Focal motor | SRSE | 11 | None in the acute phase. After 3 months diffuse cerebral and cerebellar atrophy appeared | Steroids, IVIG | Mild cognitive deficit  No epilepsy | Comatose state with anaesthesia |
| Specchio et al./ 2011 | FIRES | 4 | 6, 7, 8, 17 | 3 M, 1 F | Confusion, stupor, agitation | 6 | None | Focal motor | Responsive in 3  RSE in 1 | Und. | bilateral mesio-temporal and insular hyperintensity | Steroids, IVIG in 3 out of 4 | Mild to severe cognitive deficit/  Chronic Epilepsy | Und. |
| Gujjar et al./ 2011 | None | 1 | 39 | F | Lethargy | 4 | None | Focal motor | SRSE | 14 | bilateral mesio-temporal and insular hyperintensity then atrophy | None | Normal life  Chronic Epilepsy | Comatose state with anaesthesia |
| Ishii et al./2011 | None | 1 | 21 | M | Seizures | 7 | Mumps (PCR serum) | Focal motor | RSE | 7 | None | None | Und. | Conscious |
| Serrano-Castro et al./2013 | FIRES | 1 | 19 | F | Seizures | 7 | None | Focal motor | Responsive | 3 | bilateral insulae | Steroids | Normal life  No epilepsy | Conscious |
| Hiraga et al./2014 | None | 1 | 65 | F | Fluctuation of vigilance | Und. | Ab anti-VGKC | Focal motor | Responsive | 3 | bilateral mesial temporal hyperintensity | Steroids | Memory loss  Epilepsy outcome not reported | Spontaneous coma |
| Mumoli et al./2014 | Possible FIRES | 1 | 14 | M | Drowsiness, confusion | 10 | None | Focal occipital | Responsive | 10 | None | Steroids, IVIG | Normal life  Chronic Epilepsy | Und. |
| Cartagena et al./2014 | None | 1 | 22 | F | Und. | Und. | None | Focal motor | RSE | 7 | bilateral mesial temporal hyperintensity | Und. | Und. | Und. |
| Nair et al./ 2014 | None | 1 | 24 | F | Headache | 2 | None | Generalized TC | SRSE | Und. | None | None | Chronic epilepsy | Comatose state |
| Hwang et al. / 2014 | None | 1 | 28 | F | Seizures | 7 | None | Focal complex partial | RSE | 20 | None | Und. | Und. | Und. |
| Lapenta et al. / 2014 | None | 1 | 17 | F | Seizures | 4 | None | Focal motor; myoclonus | SRSE | 10 | bilateral hIppocampus | None | Chronic epilepsy | Comatose state |

RSE: refractory Status Epilepticus; SRSE: Super-Refractory Status Epilepticus; AERRPS: Acute Encephalitis with Refractory, Repetitive Partial Seizures; FIRES: Febrile Infection-related Epilepsy Syndrome; Und: undetermined; IVIG: Intravenous Immunoglobulin; VGKC: Voltage-gated potassium channels; TC: tonic clonic;
